# Efficient extraction of small microplastic particles from rat feed and feces for quantification

**DOI:** 10.1016/j.heliyon.2023.e12811

**Published:** 2023-01-05

**Authors:** Benuarda Toto, Alice Refosco, Jutta Dierkes, Tanja Kögel

**Affiliations:** a Department of Clinical Medicine, Centre for Nutrition, Bergen, Norway; b Department of Medical Biochemistry and Pharmacology, Haukeland University Hospital, Bergen, Norway; c Department of Biological Sciences, University of Bergen, Bergen, Norway; d Institute of Marine Research, Nordnesgaten 50, 5005, Bergen, Norway

**Keywords:** Microplastic, Extraction, Pyrolysis, Rat, Quantification, Feces, LOD, Recovery

## Abstract

To date, microplastic is ubiquitously encountered in the environment. Studies analyzing microplastic in terrestrial ecosystems, including animal feces and feed, are few. Microplastic quantification method validation and harmonization are not yet far developed. For the analysis of small microplastic, approximately <0.5 mm, extraction from organic and inorganic materials is fundamental prior to quantitative and qualitative analysis. Method validation, including recovery studies, are necessary throughout the analytical chain. In this study, we developed an optimized, efficient protocol with a duration of 72 h for the digestion of laboratory rat feed and feces. A combination of a mild acidic (H_2_O_2_ 15%; HNO_3_ 5%) and an alkaline treatment (10% KOH) dissolving the previous filter, followed by enzymatic digestion (Viscozyme®L) proved to be efficient for the extraction and identification of spiked polyamide (15–20 μm) and polyethylene (40–48 μm) from feed and feces samples from rats, showing high recovery rates. Extracted rat feces samples from an *in vivo* study in which Wistar rats were fed with feed containing microplastic were analyzed with pyrolysis-gas chromatography-Orbitrap™ mass spectrometry, quantifying recovered microplastic in rat feces in environmentally relevant concentrations. The presented three-step protocol provides a suitable, time and cost-effective method to extract microplastic from rat feed and feces.

## Highlights

•Microplastic fed to rats quantified in feces.
• Extraction of small microplastic from rat feed and feces within two days.
• Method LOD including extraction process.
• Circadian rhythm friendly protocol for HSE-compatible high throughput.
• Avoiding analyte loss by dissolving filter.

## Graphical abstract

**Figure:**
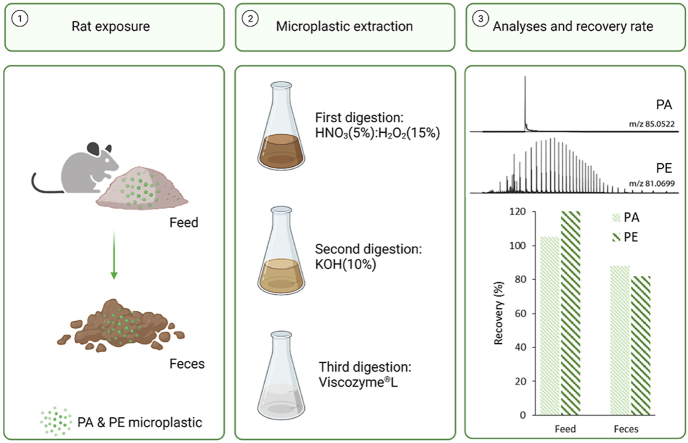

## 1 Introduction

Production of plastic has been increasing until 2019 with an unchanged volume in 2020, not taking into account the production of recycled plastic [1]. Low biodegradability and mismanagement of plastic waste are leading to increasing plastic pollution, which has become of critical concern for the environment and human health [2]. Most plastic debris persists in the environment and will eventually be fragmented into smaller particles, microplastic(s) (MP). MP are now distributed into all investigated abiotic niches, including air, soil, marine- and freshwater water surface, column, and sediment and into biota [3]. Human health might be at stake because of exposure to MP through contaminated water [[4], [5], [6], [7]] and food [[8], [9], [10], [11], [12]]. Also, feed for pets [13] and farmed fish [7,14] was reported to be contaminated with MP.

According to Prata et al. 2020 [15]; exposure to MP can produce particle toxicity in almost all biological systems leading to potential health risks such as oxidative stress, inflammation, enhanced uptake, or translocation. Furthermore, the incapacity of the immune system to eliminate synthetic particles might cause chronic inflammation and increase the risk of neoplasia [15]. Most of the studies on MP uptake and effects addressing aquatic animals reported effects such as lower food intake, reproduction, growth, dysfunction of lipid- and energy metabolism, hormone systems, nerve signals and development [16]. The few published MP exposure studies on uptake and effects in terrestrial animals, mostly mice and rats, reported effects on reproduction [[17], [18], [19], [20]], cardiovascular toxicity [21,22], metabolism [[23], [24], [25]], constipation [26] or no effects [27]. More toxic effects are reported in exposure studies with smaller MP below 10 μm, compared to larger ones in aquatic biota [16]. Moreover, the number of MP detected in the muscle and liver tissue of salmonids [14], salps [28], bivalves [16]; (overview in Table 2 of the reference) and humans [29] was higher for smaller MP below 50 μm as compared to larger ones. However, to assess the risk, exposure studies need to be evaluated in context with environmental contamination in the same size class and chemical identity. Current detection methods are insufficient to quantify MP in the small micrometre range, if not marked by rare metals, isotopes or fluorescence before exposure, and need considerable method development [30]. Recently several interesting approaches aiming at small MP quantification have been published [31,32], but recovery and proficiency tests still need to be improved and developed, respectively. Many of these approaches are developed on marine animals, and relatedly, the amount of occurrence data on marine animals is much higher as compared to terrestrial animals [10], even though the release of plastic into the terrestrial environment was estimated to be 4–23 times greater than into the ocean [33]. Lately, an increasing number of studies showed that MP are an emerging threat also to terrestrial ecosystems [34]. The occurrence of MP in feces from humans, wild animals, livestock, and pets has been documented [13,[35], [36], [37], [38], [39], [40]] but studies showing the uptake of MP into human tissues through ingestion are still scarce [20,29,[41], [42], [43]]. However, occurrence studies in human feces [38,44] and blood [45], point towards that humans ingest MP in measurable amounts and that MP can translocate across tissue barriers within the body.

As both data on the occurrence and toxicity of MP exist, a hazard is identified. However, the effects of MP on animal and human health are poorly understood and the exposure is not sufficiently quantified. Research on both aspects is a necessary step toward risk assessment.

As one step in that direction, we chose Wistar rat (*Rattus norvegicus*) as a model system for human health. This was the first mammal species domesticated for scientific research [46]. Compared to mice, rats are superior for studies on cognition and memory, breast cancer, diabetes, and mechanistic studies of human reproduction. Furthermore, the rat's body size allows for serial blood draws [47]. Dietary intake of MP would eventually lead to MP uptake in the gut, or excretion with feces. Therefore, feces may serve as a non-invasive matrix providing direct evidence of MP ingestion. One of the major technical limitations in MP quantification is the extraction from different sample matrices without affecting MP to the extent that quantifications are not possible. The digestion method must meet the following criteria: 1) effectively remove organic material from the samples so that it does not interfere with the chemical identification method while preserving the polymers, 2) facilitate the concentration of the purified samples on small pore size filters, and 3) be cost and time effective [48]. Detrimental effects on synthetic MP are observed when treated with strong acidic, alkaline, or oxidative reagents. Those methods can reduce the mass of the MP of some polymers, and these effects are different for different polymer types, introducing bias [49]. For small MP with a high surface-to-diameter ratio, this may lead to loss through the filter pores of the smallest size fractions of the most susceptible polymer types in a sample. Various extraction and analysis approaches of MP in feces of different terrestrial organisms have been published before. These range from visual identification preceded by a simple density separation step [39,50] or digestion protocol [36] to identification of plastic polymers by FTIR (Fourier-Transform InfraRed) spectroscopy preceded by more complex extraction protocols [40,51]. While microscopic individual identification of MP by identification methods such as FTIR, LDIR (Laser Direct Infrared) or Raman is meaningful for characterization of the contamination including the size distribution, it is also a time-consuming endpoint analysis [30]. For exposure control in laboratory studies or surveillance tasks requiring true quantification with a higher sample throughput, pyrolysis-gas chromatography/mass spectrometry (py-GC/MS) methods may be the better choice due to a faster analysis time of larger numbers of small particles. Samples may be pre-filtered for size separation of MP. Even though py-GC/MS does not analyze MP individually, organic and inorganic material must be removed almost completely before py-GC/MS when aiming to quantify small amounts, as otherwise the signals of other material not intended to be quantified sequester the signals from the indicator ions for the plastic polymers [52].

The goal of our study was to establish an efficient method for the removal of organic and inorganic sample constituents in rat feed and feces to enable the quantification of small amounts of MP content of different polymer types in these matrices with py-GC/MS. Initially, we tested three different digestion protocols. The best protocol was further optimized and the recovery rate of MP of physiologically relevant sizes throughout the entire purification process was determined. The digestion protocol presented here has proven to be suitable for Wistar rats’ feces and standard low-fat powdered feed and may therefore support further exposure studies. It involves a short acid and alkaline chemical digestion to efficiently degrade feces while minimizing damage to MP and an enzymatic digestion step to degrade cellulose residues. The final analysis was carried out by py-GC/MS-Orbitrap™ for chemical identification and quantification.

## 2 Materials and methods

### 2.1 Material

The following substances were purchased from the listed companies: Polyamide-Nylon (PA6) powder with a particle size of 5–50 μm (mean 15–20 μm), Goodfellow Cambridge Ltd. (Huntingdon, England); polyethylene ultra-high molecular weight (UHMWPE) powder, hereafter abbreviated PE, particle size of 40–48 μm, Sigma-Aldrich (St. Louis, MO, USA); deuterated polystyrene (PS-D_8_), Polymer Source Inc. (Dorval, QC, Canada); hydrogen peroxide (H_2_O_2_), iron (II) sulfate heptahydrate, Tween®-20, ethyl acetate and potassium hydroxide (KOH), VWR international (Radnor, PA, USA); Creon®25000 (pancreatic enzymes), Mylan (Canonsburg, PA, USA); nitric acid (HNO_3_), Viscozyme®L (cellulolytic enzyme mixture containing a variety of carbohydrases, including arabanase, cellulase, β-glucanase, hemicellulase, and xylanase), Sigma-Aldrich (St. Louis, MO, USA); Cellulase TXL, ASA Spezialenzyme GmbH (Wolfenbuttel, Germany); Whatman™ GF/D (2.7 μm pore size), GF/C (1.2 μm pore size), Cellulose Nitrate membrane filters (5 μm pore size), Cytiva (Marlborough, MA, US.). PTFE filter (polytetrafluoroethylene), BOLA (Grünsfeld, Germany); crucibles (porosity class fine, 4–5.5 μm), ROBU® (Hattert, Germany). Standard low-fat powdered feed (RM1 (E) SQC rat feed, feed content: wheat, barley, wheat feed, de-hulled extracted toasted soya, soya protein concentrate, macro minerals, soya oil, whey powder, amino acids, vitamins, micro minerals and contained 14.4% protein and 17% fibers. The fibers were mainly hemicellulose (10.2%) and cellulose (4.3%), and pectin and lignin (<2%), Scanbur (Witham, England)). Glass jars with glass lids and without plastic parts, for storage of feed, were purchased from a local household store (Clas Ohlson, Bergen, Norway). A detailed composition of all the solutions used in the experiments and of the diet is provided in the supplementary information.

### 2.2 Equipment

Digital microscope, DinoLight (AnMo Electronic Corp. Hsinchu, Taiwan); 5-digit anti-static scale (METTLER TOLEDO, XSR225 DualRange, Switzerland); high temperature laboratory oven LHT 6/60 (Carbolite Gero, Sheffield, UK); Nicolet™ Summit PRO FTIR spectrometer equipped with an Everest™ ATR accessory monolithic ATR crystal (ThermoFisher Scientific™, Waltham, MA, USA); Frontier Lab Multi-Shot Pyrolyzer™ (EGA/PY-3030D) with Auto-Shot Sampler™ (AS-1020E; Frontier Lab, Koriyama, Japan), coupled to a Thermo Scientific™ TRACE™ 1310 Gas Chromatograph with a Thermo Scientific™ TraceGOLD™ TG-5SilMS 30 m × 0.25 mm I.D. × 0.25 μm film capillary column (P/N 26096-1420; ThermoFisher Scientific™, Waltham, MA, USA), coupled to a Thermo Scientific™ Exactive™ GC Orbitrap™ mass spectrometer.

### 2.3 Ethics

The animal study was approved by the Norwegian Food Safety Authority after application through FOTS (Forsøksdyrforvaltningen tilsyns-og søknadssystem), with FOTS application ID: 20467.

### 2.4 Microplastic exposure to animals

24 Wistar rats, 12 female and 12 males, were purchased from Janvier Labs (Le Genest-Saint-Isle, France) when eight weeks old and kept alive in total for 6 weeks. The animals were divided into 4 groups and acclimatized for one week before being fed in the following way: i) control group fed with control feed (standard low-fat powdered feed, RM1 (E) SQC); ii) PA group fed with feed containing 0.1% w/w PA particles; iii) PE group fed with feed containing 0.1% w/w PE particles; iv) mixed PA:PE group fed with feed containing 0.05% w/w PE particles and 0.05% w/w PA particles. Tap water without additional treatment was provided *ad libitum*. All inlet air to animal rooms and IVC cages were HEPA-filtered. The sleeping hut inside the cages was made of porcelain and the feed was offered in porcelain plates and metallic spoons. The common, red-tinted plastic tubes normally used as enrichment in rats were replaced by ceramic items where rats can hide and better maintain the temperature in their preferred zone. The bedding material used was aspen wood and gnawing sticks were produced for animal studies supplied by Scanbur. The animals were housed for five weeks in a temperature- and humidity-controlled room (22 °C, 55 ± 10% relative humidity) on a 12 h light/dark schedule. Weight gain and well-being of the animals were controlled weekly. Then, the animals were sacrificed by carbon dioxide. Fecal matter was directly taken from the colon to avoid any contamination. For that reason, after the rat was excised, the colon was squeezed with a metallic scalpel, and the collected feces were stored in aluminum foil at −80 °C.

### 2.5 Preparation of the feed

Standard low-fat powdered feed is a high-quality human food grade soybean concentrate with a low protein and nutrient level. The diet was prepared in batches of 1 kg, and the same steps were followed for each batch. Standard low-fat diet in powder was carefully mixed in a volumetric way with MP 1:1000 (w/w) under a fume hood. To ensure homogenization, feed powder and MP were mixed 1:1 (w/w) in a separate glass container with a glass lid. The mixture was blended by shaking the container vigorously. The procedure was started with 1 g:1 g and then consecutively doubled amounts of feed were added in increments up to 128 g. Then the mixture of a total of 256 g was blended in a Bosch Universal plus 800 W stand mixer on the lowest setting. The remaining 744 g of feed were carefully added into the mixer, which was then set to the highest setting for 5 min.

### 2.6 Preparation of feces

#### 2.6.1 Protocol development for microplastic isolation

Three different protocols were tested for their efficiency to digest feed and feces samples.

##### 2.6.1.1 Protocol 1

Protocol 1 is based on treating the samples using Fenton's reagent as described by Yan et al. 2020 [53]. Briefly, Fenton's reagent was composed of 30% H_2_O_2_ and an iron catalyst solution prepared with 10 g iron (II) sulfate heptahydrate in 500 ml Milli-Q water. The iron catalyst solution and H_2_O_2_ were added into glass beakers in sequence according to a 1:2.5 ratio (v/v). Samples of feed and feces were placed in 150 ml Erlenmeyer flasks and Fenton's reagent was added as described by Yan et al. 2020, incubated at pH 3, at 15–40 °C for 4.5 h. The solutions were then diluted at 1:2 ratio (v/v) with Milli-Q water and filtered through Whatman™ GF/D (2.7 μm pore size).

##### 2.6.1.2 Protocol 2

In protocol 2, feed and feces samples were treated with KOH, as previously described for dissolving soft tissue of biota for MP analysis [49]. Samples were placed in 50 ml crucibles with fine ceramic filters (pore size: 4–5.5 μm) and incubated with 10% KOH for 1, 2 and 5 days at 40 °C.

##### 2.6.1.3 Protocol 3

Protocol 3 is based on three digestion steps: i) acidic digestion with HNO_3_ and H_2_O_2_ [54]; ii) alkaline digestion with KOH and iii) enzymatic digestion. Feces and feed samples were initially incubated for 2, 6 or 24 h at 40 °C with 15% H_2_O_2_/5% HNO_3_. The digestates were vacuum filtered through 5 μm cellulase nitrate filters, followed by optimization of time and temperature. Cellulose nitrate filters were chosen for efficiency and improved recovery, since these could be dissolved in the next step in 10% KOH without an additional step.

Thus, in the second step 10% KOH was added to the flasks containing the filter and incubated under the optimized conditions. After one day the samples were filtered through 5 μm PTFE filter.

In the third step, three types of enzymes were tested as follows: **Protocol 3A**: Creon25000®, a pharmaceutical product that contains three different pancreatic enzymes: lipase, amylase and protease, previously used for dissolving soft tissue of mussels by von Friesen et al. 2019 [55]. The content of one Creon25000® capsule was firstly dissolved in Tris hydrochloride (HCl) solution (pH 8, 1 M) and then added to the samples. Next, samples were incubated for 24 h at 40 °C, 140 rpm**. Protocol 3B**: Viscozyme®L, a cellulase mixture also sold for commercial food plant extraction. Viscozyme®L, has previously been used for MP analysis in soils samples [56] and in a master thesis for street dust samples and sediments [57], but not for biota. Viscozyme®L was added to the samples together with NaOAc (C_2_H_3_NaO_2_) buffer solution (pH 5, 1 M) and incubated for 24 h at 40 °C. **Protocol 3C**: Cellulase TXL, a commercial cellulase was added to the samples together with NaOAc (C_2_H_3_NaO_2_) buffer (pH 5, 1 M) and incubated for 24 h at 50 °C, 140 rpm.

The digestates were filtered through Whatman™ GF/C (1.2 μm pore size) and placed into glass Petri dishes with lids in an incubator at 56 °C to dry prior py-GC/MS-Orbitrap™ analysis.

Erlenmeyer flasks and samples in each digestion step were rinsed with 0.01% (m/m) Tween®-20, ethanol-water (1:1, v/v), and water. In the final filtration step the filters were rinsed with pre-filtered 0.01% (m/m) Tween®-20, ethanol-water (1:1, v/v), and 96% ethanol. Lastly, a few drops of 20% KOH were added directly on the filter to facilitate the filtration.

A detailed version of the optimized protocol (protocol 3B) with all the steps included is provided in the supplementary information.

Undigested material after the different digestion steps was analyzed by ATR-IR spectroscopy. A Nicolet™ Summit PRO FTIR spectrometer equipped with the Everest™ ATR accessory monolithic ATR crystal was used to collect spectra from 4000 cm^−1^–440 cm^−1^. For each sample 16 co-added scans with spectral resolution of 4 cm^−1^ were collected and 32 pre-recorded background scans were used for correction. Spectra were processed in the OMNIC Paradigm Desktop software (ThermoFisher Scientific™, Waltham, MA, USA) and compared to spectra of known compounds and polymers in commercial libraries (Thermo Scientific™), open-source libraries (simple-plastics.eu [58]; and in-house libraries of plastic and natural polymers.

#### 2.6.2 Microplastic identification

MP were analyzed at the Chemistry & Undesirables Laboratory at the Institute of Marine Research, Bergen, Norway. Py-GC/MS-Orbitrap™ was used to quantify the mass of polymers found in the controls and spiked samples. The mass spectrometer was operated in full-scan mode using 60,000 mass resolution (measured as FWHM at *m*/*z* 200). Samples were transferred to stainless steel pyrolysis cups with deactivated silica surface and added 20 μm 1.8 10^−1^ μM PS-D_8_ (deuterated polystyrene) in ethyl acetate. After drying, the cup was injected into the pyrolyzer at a constant temperature of 700 °C. Samples were pyrolyzed for 1 min, and the pyrolysis products were injected into the GC with a spilt of 20. Temperature in the GC was increased by 15 °C min^−1^ and held at 320 °C for 15 min. Quantification/qualifier ions (PA: 113.0835/85.0522; PE: 81.0699/67.0542) were selected based on literature data and customized libraries [59]. Method specific limit of detection (LOD) was calculated for each polymer from blank samples [60]. Limit of quantification was set to 3.3 × LOD [60].

#### 2.6.3 Contamination avoidance and monitoring

The possibility of contamination in the laboratory was reduced by always wearing cotton lab coats and nitrile gloves during sample processing, digestion and analysis. Plastic free equipment (i.e., metallic tweezers, spoons, and spatula) was used in all steps and was carefully cleaned between samples using filtered water. Furthermore, all tools and glass equipment were burned prior to use in a muffle oven at 500 °C for at least 5 h, and covered by aluminum foil throughout preparation and digestion, when possible, to minimize airborne MP contamination. The surgical tools used to dissect the rat and squeeze the feces from the colon without opening it were cleansed in soap and water before being autoclaved. All chemical solutions were stored in glass containers covered by glass lids. Solutions used, including enzymatic solutions, were prepared with Milli-Q water and pre-filtered with Whatman™ GF/C.

Additionally, all filters were tested for chemical damage. For that purpose, chemical solutions were vacuum filtrated through, rinsed with Milli-Q water and observed under a digital microscope DinoLight (AnMo Electronic Corp. Hsinchu, Taiwan). To avoid MP contamination from the cage or the fur of the animals, feces were directly extracted from the gut, wrapped into aluminum foil and frozen at −80 °C until analysis.

Thus, the most efficient protocol (**protocol 3B**) for the extraction of MP from the feces was further evaluated by performing blank and negative control experiments in triplicate. Blank control was performed without matrix, to evaluate procedural and background contamination. Negative control was performed with feed and feces samples without added MP. Positive controls were conducted in triplicate: feed and feces samples were spiked with a known amount of both PA and PE particles and subjected to the optimized protocol. Recovery rates of MP were analyzed.

### 2.7 Statistical analysis

The collected data did not follow a normal distribution pattern with the Kolmogorov-Smirnov test (KS). Kruskal-Wallis 1 way ANOVA test for multiple comparisons was performed to determine the significance of the differences of the PA and PE content in feed and feces between negative and positive control experiment and among PA and PE content between four exposure groups. All data were analyzed using the SPSS© Statistic package version 26 (IBM©). For all statistical analysis, the level of significance was set at p < 0.05 to allow for 95% confidence limit. All data are expressed as the mean ± standard error of the mean (SEM).

## 3 Results

### 3.1 Animal study

The rats were fed with feed containing either 1) no MP, 2) PA, 3) PE or 4) their mixture for five weeks at a dose of 0.1% w/w in total. The feed was well tolerated, and no symptoms of suffering or distress were identified during the trial. Body weight had a positive trend in all exposed groups and was indistinguishable among the groups. The rats were sacrificed after five weeks of exposure and the gut was excised. Fresh feces were collected directly from the colon as described, avoiding any contamination of the feces by MP from the cage or fur. The results of the rat exposure effect study were published recently [61].

### 3.2 Method development and validation steps for microplastic analysis

#### 3.2.1 Initial tests

To develop a protocol to efficiently extract well-preserved MP from rat feed and feces, initial experiments were performed to determine the optimal type and order of the regents used, incubation time, shaking pulse, temperature, type of filters used. Each experiment was carried out in triplicate. Several attempts were also done using enzymes only. Different types of commercial enzymes, such as amylase, lipase and protease, were combined with different incubation times of 24–72 h. However, the digestion of our matrices was not effective enough with these types of enzymes and the samples could not be filtered efficiently at pore sizes ≤5 μm. As the activity of the enzymes decreases with time, it was also attempted to use fresh enzymes for three consecutive days without success. It should also be noted that processing larger samples would result in unacceptable cost because of the price of enzymes. Eventually, this method was abandoned due to the high costs of the enzymes and the many steps, leading to a higher risk of sample loss to equipment and contamination. However, an additional enzymatic digestion step after acidic-oxidative and alkaline digestions was then considered as potentially less costly, as the samples would be pre-digested, decreasing the amount of enzyme needed. A combination of acidic-oxidative and alkaline-chemical digestion followed by enzymatic treatment (**Protocol 3**) was the most efficient protocol for MP extraction.

#### 3.2.2 Digestion protocols

**Protocol 1** (Fenton reagent): This protocol was tested with 1 g of sample as described by Yan et al. 2020 [53]. High amounts of undigested material present on the filters led to slow filtration rates of 1.5 h and 2 h for the feed and feces, respectively. Excessive foam formed during digestion which was minimized by adding a mixture of ethanol and water in a ratio of 1:1. Even with the additional step to prevent excessive foaming, the filtration process took a long time and needed more than one filter to filter the whole sample. Thus, we regarded as inacceptable for reaching a meaningful sample-throughput. Digestion efficiency, calculated as described by Karami et al. 2017 [62]; was <90%. Therefore, this protocol was not further optimized.

**Protocol 2** (10% KOH): Incubation for one or two days was insufficient to properly digest the matrices, whereas five days of incubation resulted in damaged ceramic filters of the crucibles (ROBU® 50 ml crucibles; fine), to the extent of visible degradation and MP trapping at the interface between ceramic filter and glass wall. Therefore, and because of excessive duration, protocol 2 was not further optimized.

**Fig. 1:**
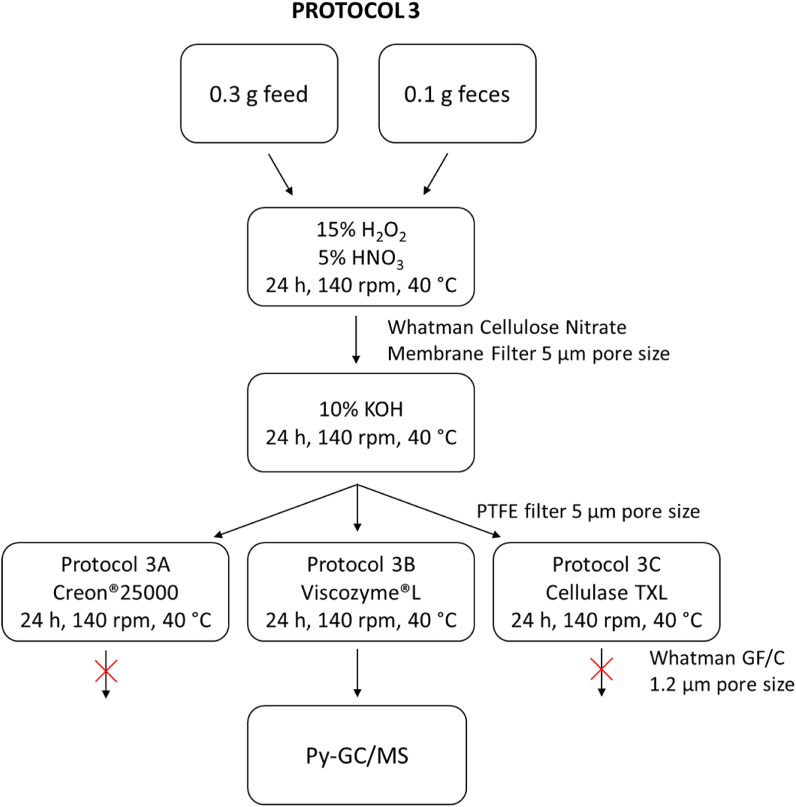
Flow diagram that illustrates the main development steps of the optimal protocol 3B.

**Protocol 3** (combined chemical and enzymatic digestion): The combination of acidic-oxidative and alkaline digestion resulted in a higher digestion efficiency as compared to the Fenton's reagents and KOH alone, observed visually, for both feed and feces. This protocol was then further improved with an enzymatic digestion step. The material on the filter after Creon® enzyme treatment was treated with an acidic-oxidative and alkaline step and identified as cellulose by ATR-IR analyses (see supplementary material, **Figure S1**). Therefore, two commercially available cellulases, Cellulase TXL (**protocol 3C**) and Viscozyme®L (**protocol 3B**), were compared to Creon® as last digestion step, consecutive to acidic-oxidative and alkaline digestion. When samples were treated with Cellulase TXL the filters clogged completely, and filtration was not possible (**Figure S2 a, b**). For samples treated with Creon® or Viscozyme®L the filtration was feasible (**Figure S2 c**). Following treatment with Creon®, there were more remaining undigested materials on the filters, as compared to Viscozyme®L, and more then what could be analyzed by py-GC/MS. Furthermore, there was more of the white fibrous mass left of fecal than from diet samples, suggesting that this substance might be concentrated by the rats in the feces. ATR-IR of this substance suggested a mostly homogenous mass of cellulose species. In summary, of all the enzyme tested, Viscozyme®L led to the best digestion efficiency and filtration rate and was therefore chosen for further optimization (Fig. 1).

#### 3.2.3 Rinsing

For **Protocols 3 A and 3 C**, samples were rinsed with Milli-Q water only, to investigate potential interference between consecutive reagents. This was compared to detergent-ethanol-mix and consecutive water (see methods section, **protocol 3**).

**Fig. 2:**
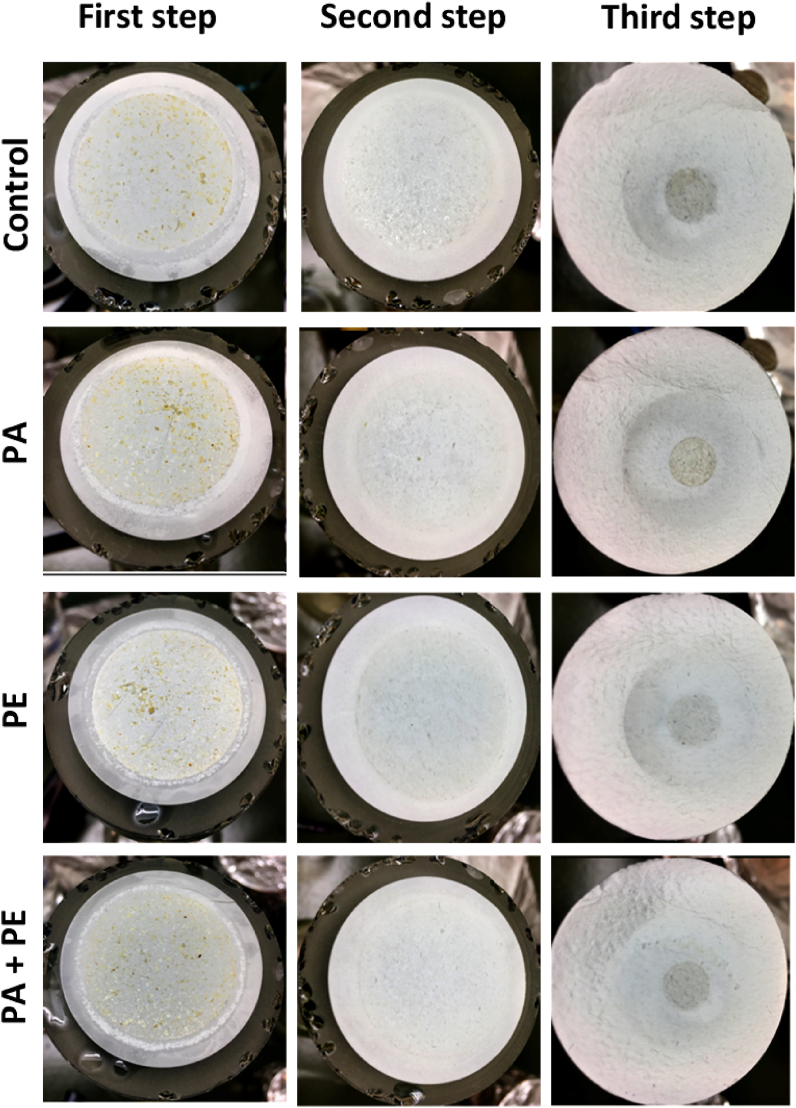
Filters during digestion of feces samples from rats using the optimal **protocol 3B**. First step: remnants of feces material on Whatman™ Cellulose Nitrate Membrane filters; second step: remnants of feces material on PTFE filters; third step: remnant of feces material on Whatman GF/C™ filters. In the third step, a thinner filtration setup column to center the remaining residues was used. The filter was then cut and folded properly to fit inside the pyrolizer cups.

It was observed that when only rinsing with water, filtration time was long, especially in the last step of digestion where samples were transferred to the filters with the smallest pore size. Several washing steps were performed separately and combined until we settled on the use of Tween®-20, ethanol-water (1:1, v/v), water after the oxidative-acidic and alkaline treatment, and Tween®-20, ethanol-water (1:1, v/v), 96% ethanol in the final filtration step. The application of these washing procedures in the above-mentioned order to rinse samples eased the filtration process and the recovery of MP from the glass container, avoiding clogging possibilities (Fig. 2).

#### 3.2.4 Incubation time and temperature

Initially, in **protocol 3** we tested three different incubation times (2 h, 6 h and 24 h) for 15% H_2_O_2_/5% HNO_3_ treatment at 40 °C and two different temperatures (40 °C and 50 °C) for enzymatic treatment. For better digestion efficiency and filtration rates, the optimized incubation time was found to be 24 h, as the shorter incubation times did not lead to a complete digestion of the organic matter present in feed and feces samples.

Regarding the incubation temperature no difference was noted between 40 °C and 50 °C. Both were equally good for the Viscozyme®L activity. To better preserve the integrity of the plastic polymer and to save energy, an incubation temperature of 40 °C was chosen in the optimized protocol.

#### 3.2.5 Filters

With the filters used in protocol 1, Whatman™ GF/C and GF/D filters, with 1.2 and 2.7 μm–very small pore sizes, the filtration was unsuccessful for 1 g samples of feed or feces. To avoid filter clogging, filters needed to be changed during the filtration of each sample. A metallic micro spoon-spatula was used to scrape off the clogging material from the filters and to transfer it into the Erlenmeyer flask for the following step. Some of the material got stuck in the filters leading to material loss. Small pieces of the filters were ripped off during the scraping procedure, thus contaminating the sample. Due to concerns of unreliability, this filter type was not further used.

The filters used for protocol 2, ROBU® 50 ml crucibles Por. fine, are made of pure borosilicate glass 3.3. Even though they are characterized by being highly resistant to most chemicals and temperatures up to 500 °C, five days of treatment with KOH 10%, at 40 °C, 125 rpm led to structural damage of the crucible filters. Compared to other filters used in this study, these filters are thicker and small particles become more frequently entrapped in the filter material, causing sample loss. Hence, ROBU® 50 ml crucibles Por. fine filters were not further used.

Whatman™ Cellulose Nitrate Membrane Filters, 5 μm, were used for protocol 3 for filtering the samples after treatment with H_2_O_2_ 15%/HNO_3_ 5%. They were easy to handle and using them avoided sample loss as compared to the other tested filter types. They are strong, flexible and have a low extractable level (material released from the filter) according to the manufacturer's specifications (https://www.sigmaaldrich.com/DE/en/product/aldrich/wha10400214). Their larger pore size of 5 μm is an advantage, as it allows a faster filtration. It should be noted that PA particles used in this study have a size of 5–50 μm (mean 15–20 μm) and two of the filters used (Whatman™ Cellulose Nitrate Membrane Filters and PTFE filter) have a pore size of 5 μm. Therefore, the smallest PA particles (5 μm) could be lost during the filtration process. However, comparable recovery rates of PA and PE particles in feed and feces samples indicate that the loss was negligible, at least in terms of mass.

Furthermore, Cellulose Nitrate filters dissolve completely in 10% KOH, facilitating the consecutive alkaline digestion step, and thereby preventing loss of MP, as the transferring process of the sample to the next step is unnecessary.

PTFE filters, 5 μm, were chosen due to their hydrophobic nature, chemical stability and inertness, proven to be resistant to aggressive solvents and alkaline solutions.

Finally, residual samples were transferred to Whatman™ GF/C filters (1.2 μm pore size) and were prepared for py-GC/MS-Orbitrap™ analysis. In this final step, most organic material is already removed and consecutive filtering is compatible with the pore size of 1.2 μm.

## 4 Quality assurance/quality control

Except from crucibles used in **protocol 2** and Whatman™ GF/C or GF/D filters, 1.2 and 2.7 μm, respectively used in **protocol 1**, none of the filters used in **protocol 3** showed signs of chemical degradation or damage.

Blank samples (water) were subjected to the entire extraction procedure. Filters obtained in the end did not reveal contamination when analyzed with py-GC/MS-Orbitrap™. Ten of these samples were used to calculate the method specific limit of detection (LOD), which is specific for each polymer [60] (Table 1). The limit of quantification (LOQ) was set to 3.3 × LOD [60]. Negative control showed presence of PA and PE below the LOD of the py-GC/MS-Orbitrap™ (Table 1).

**Table 1:** **Results of the negative control experiment.** Mean values of MP detected in feed and feces samples without added MP. All data of the matrix controls are expressed as the mean ± standard error of the mean (SEM), n = 3.

	PA (μg/g sample)	PE (μg/g sample)
Results procedural controls (n = 10)		
LOD	1.4	11.8
LOQ	4.5	38.9
Results matrix controls (n = 3)		
Feed	4.4 ± 0.9	9.1 ± 1.6
Feces	3.9 ± 2.3	6.0 ± 3.2

**Table 2:** **Percentage recovery of PA and PE MP from spiked feed and feces samples.** All data are expressed as the mean percentage ± standard error of the mean (SEM); n = 3.

	Recovery rate PA (%)	Recovery rate PE (%)
Feed	105 ± 31	121 ± 66
Feces	88 ± 14	82 ± 28

The positive control experiment performed with feces and feed samples spiked with both PA and PE showed good recovery rate (Table 2).

Kruskal-Wallis one-way ANOVA test for multiple comparisons for differences of the PA and PE content in feed and feces between negative and positive control experiment showed significant differences (p < 0.05) in all cases.

## 5 Microplastic in feces

Initial tests with μFTIR confirmed the presence of both PA and PE in feed and were further specified by the results of the analysis with py-GC/MS-Orbitrap™. Since the rats were only fed this feed and gained weight normally, this confirmed that MP were ingested by rats. The results of feces analysis with py-GC/MS-Orbitrap™ further demonstrated that MP were excreted by rats.

The fecal material of the rats within each of the four different groups was pooled (two rats from control and four rats from each MP group) and then homogenized to minimize the variability of the individual sample. Then extraction according to protocol 3 B was carried out in triplicates.

Results showed that MP were detected in the feces of all the three exposed groups, PA group, PE group and mixed PA + PE group, while no PA or PE above the LOD was detected in the control group. The concentrations of MP in the feces for the group exposed to PA and the group exposed to PE were similar, 0.76 ± 0.17 mg/g and 0.66 ± 0.06 mg/g per sample respectively. Whereas 0.33 ± 0.09 mg/g PA and 0.42 ± 0.01 mg/g PE were detected in the group exposed to both PA and PE, no MP were found in the control group (Table 3).

**Table 3:** **MP detected in feces in the exposure experiment.** Average values of MP detected in feces samples. Results are reported as the mean of the three replicates ±SEM.While.

	PA (mg/g)		PE (mg/g)	
	Triplicate	Average	Triplicate	Average
Control group	<LOD	<LOD	<LOD	<LOD
	<LOD		<LOD	
	<LOD		<LOD	
PA group	0.42	0.76 ± 0.17	<LOD	<LOD
	0.99		0.014	
	0.88		<LOD	
PE group	<LOD	<LOD	0.54	0.66 ± 0.06
	<LOD		0.71	
	<LOD		0.73	
PA + PE group	0.27	0.33 ± 0.09	0.40	0.42 ± 0.01
	0.50		0.44	
	0.22		0.41	

The collected data did not follow a normal distribution pattern (KS test). Therefore, a non-parametric Kruskal–Wallis one-way ANOVA test for multiple comparisons were performed to determine the significance of the differences between PA and PE content among four exposure groups (Table 4).

**Table 4:** **P-values obtained from the comparison of the exposure groups.** Results of Kruskal-Wallis tests for multiple comparison. * *p*-Values <0.05.

PROTOCOL 3B		
Group comparisons	PA feces	PE feces
Control to PA	0.009*	0.496
Control to PE	1	0.004*
Control to Mix (PA + PE)	0.08	0.061
PA to PE	0.009*	0.027*
PA to Mix (PA + PE)	0.398	0.234
PE to Mix (PA + PE)	0.08	0.307

There were statistically significant differences in MP detection in feces samples between the control and PA exposed group, the control and PE exposed group, and between the PA exposed group and the PE exposed group (Table 4). The differences between the control and the mixed group, which had half the concentration per analyte as compared to the PA and PE group, did not reach statistical significance, likely due to the lower concentration and small sample size.

## 6 Discussion

Detection and analysis of MP in feces samples is likely to be a useful indicator for the estimation of MP exposure and ingestion in different animals as well as humans in a non-invasive way. For analyzing health effects on humans, model systems need to be developed. Food safety studies heavily rely on mammalian animal model systems, which are required for the setting of many estimations and limits with legal impacts, such as EFSA tolerable intake estimations, considered for trade limits such as EU Maximum levels and recommendations from food safety authorities. Yet, most studies that so far have analyzed feces and feed were done with terrestrial and aquatic animals, such as seabirds [36], (*Callorhinus ursinus)* [37] and pet animals (cat and dog) [13]. In order to be reliable, the analysis methods need to be tested and potentially developed specifically for the species and matrix in question. To analyze compliance with maximum levels methods with defined measurement uncertainties and method limitations including the sampling and extraction procedure, need to be developed. To optimize our protocol, different extraction approaches were compared.

Fenton's reagent treatments tend to lead to excessive foaming, which might lead to sample loss. This had been discussed by others before [[63], [64], [65]]. On the other hand Yan et al. 2020, proposed a novel approach tested on human, chicken and zebrafish feces with high digestion efficiency that involves Fenton's reagent, nitric acid, and ethyl alcohol to remove feces solids [53]. Therefore, we included this as protocol 1 for comparison into this study.

10% KOH alone is widely applied for digestion of material rich in proteins as they hydrolyze in alkaline environments [66]. However, it was abandoned for our purposes due to long duration and filter degradation. Excessive duration of three weeks in some cases, was also discussed and abandoned by others for large samples and monitoring programs with large sample numbers [67]. Additionally, further development was necessary for adaptation to feces as sample matrix, as animal tissues and feces have different compositions. While animal tissues are dominated by protein, feces solids (25%) are composed of 25–54% bacterial biomass, 25% carbohydrates or any other non-nitrogenous undigested plant matter, 2–25% protein or nitrogenous matter and 2–15% undigested lipids, resulting in domination of polysaccharides and fibers [68]. The main difference relevant for the digestion is the plant-derived content [69]. Prata et al. found that H_2_O_2_ worked better for plant material, while KOH worked better for animal tissue. Therefore, we tested acidic-oxidative digestion followed by alkaline digestion. As the remaining material was cellulose, we used enzymatic treatment to fully digest the sample. In the first step, 15% H_2_O_2_/5% HNO_3_ was tested. HNO_3_ was published before to be an efficient oxidant of organic matter [70,71], and combining it with H_2_O_2_ to increase reaction speed [54,72,73]. The corrosiveness of HNO_3_ and KOH in combination with high temperature (>40 °C) against PA has been previously confirmed with decreased recovery rates [63] and change of color [62,74]. To avoid that to the extent possible, it is essential to choose the optimal concentration of HNO_3_. In our protocol, a concentration of 5% HNO_3_ in combination with 15% H_2_O_2_ was used to digest the organic content of the samples, based on [54]; who reported a high recovery efficiency (98%) for PA and PE at 40 °C for 12 h. However, in our study, a longer incubation time of 24 h led to a better digestion of the organic material, and a recovery rate for PA of 105% ± 31. It is worth mentioning that the exposure time of 24 h used at all steps of our protocol is a key factor to make the protocol circadian rhythm friendly for the laboratory personnel.

Regarding the last step, enzymatic digestion was often preferred compared to other chemical treatments due to their negligible impact on the plastic polymers and laboratory safety [66,75]. Since 2014, enzymes have been used regularly in digestion protocols due to their capability to target specific biological materials as diverse as plankton-rich seawater samples [66], mussels [76] and bivalves [55]. Cellulase TXL was previously successfully used, in combination with other enzymes to digest marine plankton samples [48]. However, on our matrices, Cellulose TXL proved to be inconsistent and inefficient. This may be caused by the temperature we used (40 °C) which might have led to a reduction in the enzymatic activity compared to 50 °C used by Loder er al. 2017 [48]; even though the temperature range of the enzyme is 35–60 °C. Furthermore, according to Loder et al. 2017; this process should be done three times with a larger concentration of enzyme each time, and the cellulase solution should be refreshed every 24 h to obtain a high digesting efficiency, causing large expenses both in laboratory time and enzyme cost. Viscozyme®L was previously used to purify soil samples in combination with other three enzymes in a seven-steps (7 days) enzymatic-oxidative digestion protocol (order: SDS, Fenton reagent, Protease, Pectinase, Viscozyme, Cellulase, Fenton reagent [56]) and for street dust samples and sediments [57]. Despite high purification efficiencies, a 7-day long protocol might not be suitable for analysis of large numbers of samples. In the present study, following chemical pre-digestion, Viscozyme®L was incubated at 40 °C for 24 h in comparison to the incubation of 48 h at 50 °C used by Möller and co-authors. This might have been enabled by the chemical pre-digestion steps. It led to a significant and sufficient decrease in the amount of non-digested material on the filter and was therefore applied in that way in the optimized protocol. Most enzymatic digestion methods currently available use several enzymes with a long incubation time to fully digest the matrices. A long incubation time can decrease the optimal activity of the enzymes. Furthermore, a higher number of processing steps increases the cost, the risk of procedural contamination and sample loss, subsequently decreasing the accuracy of the method. The cost of enzymes is among the largest monetary posts in digestion protocols for MP analysis, especially when large samples are used. For this protocol, few digestion steps are needed and only one enzyme is used.

Another aspect to consider is that the pore size of the filters chosen has a fundamental effect on the MP extraction [77]. Particles smaller than the pore size will be lost, but a small pore size is not necessarily better, they can lead to clogging with some matrices, as observed in this study (protocol 1).

Our results of the application of the optimized protocol on feces samples from a rat exposure study showed that the concentrations of PA and PE in rat feces reflected the concentration ratios present in the feed. The diet administered to rats in the present study contained 1 mg/g MP, one order of magnitude higher than the estimated presence of MP in normal pet feed and fishmeal, around or below 100 mg/kg [7,13]. The amounts of plastic detected in the feces of our exposed rats ranged between 0.3 mg/g and 0.7 mg/g. The detected similar range of concentration of the MP in the feces as compared to the feed is in agreement with previously reported limited uptake of MP through the gastrointestinal tract in the low to sub-percentage levels [[78], [79], [80]].

We here present a method LOD and LOQ, which takes into account the entire sample preparation, as is required for comparisons with toxicity testing. To the best of our knowledge, this is the first study quantifying MP in laboratory rat feed and feces presenting a method LOD and LOQ.

Two major challenges of MP extraction and identification are a) underestimations due to sample loss during the procedure and b) overestimation caused by contamination. Therefore, quality assurance and quality control are vital to assess the methods along the whole procedure of MP sampling, extraction and identification [81]. Indeed, despite many precautionary measures implemented to minimize the procedural contamination as described above, PE was detected in our control and PA groups, but below the level of quantification. This might derive from cross- or airborne contamination during the preparation of the feed or the processing of the samples. The PE used in our study was more difficult to handle than PA, as they had higher static electricity. This phenomenon was also observed by Catarino et al. 2017 [75] for nylon. It has a lower density of 0.94 g/ml at 25 °C and a higher surface resistivity 10^13^ as compared to PA, which had a density of 1.13 g/cm^3^and 5 × 10^10^ (Ohm/sq), respectively (http://www.goodfellow.com (2021)). This points towards PE being a good insulator while PA may accumulate positive charge and have certain anti-static properties. Such differences may lead to polymer-type dependent bias in analysis results and need to be controlled for with polymer type specific limit and uncertainty calculations.

To date, to our knowledge, only one other study on feces digestion methods on terrestrial organisms has performed recovery testing of the protocol used (Table 5 [36,[38], [39], [40],^50^,^51^,^53^]) [53]. used H_2_O_2_ as part of the Fenton reagent followed by two consecutive digestion steps with HNO_3_ alone and at shorter incubation time ((1) HNO_3_ 65%, 50 °C for 30 min and (2) HNO_3_ 65%, 70 °C for 10 min). Since we wanted to analyze polyamide, which, according to Prata et al. 2019 [69]; is particularly susceptible to acids and may deteriorate especially at high concentrations and high temperatures, we chose a milder protocol [53]. obtained a recovery rate of more than 97% which was higher than our values for feces recovery (82–88%). However, the size of MP particles used for the recovery testing was larger than the size of the particles investigated in our study and the particles tested for digestion effects on the particles was even larger (Table 5). Most studies that have conducted recovery experiments and obtained high recovery rates have tested manufactured particles larger than the size range of the MP being analyzed [38,53,62]. Most of them proved no visual or chemical change in the properties of the plastic, some found a small change in the weight of the particles. While a mass loss of <0.5% in bigger particles (1–4 mm) can be considered acceptable for an extraction method, the same is not valid for smaller particles as the surface-to-volume ratio increases with decreasing size, which will lead to increased damage to the chemical integrity of the particle [82]. The size of the particles used may affect their recovery, also as smaller particles are more likely to adhere to the surfaces of the glass containers and the vacuum filtration unit due to a greater surface charge resulting from their higher surface-to-volume ratio [83]. To arrive at correct uncertainty estimations and particle losses, recovery studies need to be size and polymer type specific. Future studies should test recovery on particles comparable to their analytes in size and polymer type, and an organic matrix should be used instead of clean aqueous solutions, due to potential matrix effects. In our experiment, the MP used for the recovery experiment were the same particles used in the exposure study and underwent all the steps of the analysis. The results of the spiking experiments presented in our study showed that the multiple digestions did not cause significant loss of the analyzed MPs.

**Table 5:** Overview of studies on feces digestion in terrestrial organisms.

Matrix **Reference**	Extraction method, MP identification method	MP size range and LOD	Polymers identified	Recovery analysis MP for recovery analysis
Feces (chicken) [50]	Density separation; Visual inspection, heat test	0.1–1 mm	NA	NI
Feces (sheep) [39]	Density separation; Visual inspection, heat test	NI	NA	NI
Fecal precursor (seabird) [36]	30% H_2_O_2_:RT, 24 h/75 °C, 8 h; Visual inspection	100–1000 μm	NA	NI
		LOD: 100 μm		
Guano (penguin) [51]	10% KOH, 40 °C, 24 h/10% H_2_O_2_, 24 h; μ-FTIR	76–4945 μm	PE, Rayon, PAN, PP, PES, PAA	NI
		LOD: 60 μm		
Feces (human) [40]	30% H_2_O_2_, 20 days; FTIR	20–800 μm	PP, PET, PS, PE. PVC, PC, PA, PU	NI
		LOD: 10 μm		
Feces (human) [38]	30% H_2_O_2_, 25 °C, 2 weeks/0.05 M NaOH, 25 °C/Imidazolium salt, 25 °C; FTIR	50–500 μm	PP, PET, PS, PE, POM, PC, PA, PVC, PU	Damage tested, no recovery analysis. 0.2–1 mm, same polymer types as analytes
		LOD: 50 μm		
Feces (human, chicken) [53]	Fenton's reagent 5 h, <40 °C/65% HNO_3,_ 50 °C, 30 min/70 °C, 10 min/ethanol; Raman	LOD: >1 μm (Filtration pore size, Raman resolution)	Chicken (C): PET	H: 100% PE 150 μm, PS 250 μm; 93,33% PVC 75 μm. C: 100% PE; 96.67% PS; 97.78% PVC. Digestion tested on 2–4 mm MP.
			Human (H): PBT, PVB	

FTIR: Fourier-transform infrared spectroscopy; LOD: limit of detection; NA: not applicable; NI: not investigated; PAA: polyacrylate; PA: polyamide; PAN: polyacrylonitrile; PBT: polybutylene terephthalate; PC: polycarbonate; PE: polyethylene; PES: polyester; PET: polyethylene terephthalate; PMMA: polymethylmethacrylate; POM: polyoxymethylene; PP: polypropylene; PS: polystyrene; PU: polyurethane; PVB: poly (vinyl alcohol-*co*-vinyl butyral); PVC: polyvinyl chloride.

### 6.1 Limitations

This method allowed efficient digestion of rats' feed and feces; however, the samples were small and the applicability to larger samples might not be efficient. This laboratory-based study oversimplifies the natural conditions where the diet can be variable. In the present study, a cellulase has been used as an adequate component to break down cellulose, as the diet used to feed rats had a higher content of fibers, and consequently a higher amount of cellulose. However, there is a high diversity in diet composition among the species that could impact the applicability of protocols involving an enzymatic digestion. Therefore, the enzymes used in the method need to be adapted based on the food preference. It is, therefore, necessary to test this aspect for applicability to environmental samples. Unfortunately, a generalized protocol for different sample matrices is still not developed. Furthermore, the MP used in the present study have well-defined shapes, sizes, and polymer types, while MP found in the environment are heterogeneous. Therefore, this method needs to be verified for a broader range of plastic polymer sizes.

## 7 Conclusions

In the present study, we developed a three-step cost- and time-effective digestion protocol which digests rats' feed and feces samples sufficiently for MP analysis. The developed method was successfully applied to the analyses of MP in rats’ feces with good recovery rates for two different polymer types and circadian rhythm friendly for the laboratory personnel. To the best of our knowledge, this is the first study quantifying MP in laboratory rat feed and feces presenting a method LOD and LOQ.

## Contributions

BT, AR, JD and TK conceived and designed the experiments, contributed reagents, materials, analysis tools or data and wrote the paper; BT and AR performed the experiments.

Tanja Kögel reports an advisory committee relationship with the Netherlands Organization for Health Research and Development (ZonMW).

## Appendix A Supplementary data

Revised version with track changes_2 Revised version with track changes_2
