# Supplementary material for: Efficient extraction of small microplastic particles from rat feed and feces for quantification
Source: Heliyon. 2023 Jan 5;9(1):e12811. doi: 10.1016/j.heliyon.2023.e12811 (PMC9876835; doi:10.1016/j.heliyon.2023.e12811)
Supplement: Revised version with track changes_2 [file mmc1.docx]

Supplementary information for:

# Efficient extraction of small microplastic particles from rat feed and feces for quantification

Benuarda Toto^1, 2^, Alice Refosco^1, 2^, Jutta Dierkes^1,3^ and Tanja Kögel ^4,5, 6^

1: Department of Clinical Medicine, Centre for Nutrition, Bergen, Norway; 2: first two authors contributed equally. 3: Department of Medical Biochemistry and Pharmacology, Haukeland University Hospital, Bergen, Norway; 4: Department of Biological Sciences, University of Bergen, Norway; 5: Institute of Marine Research, Nordnesgaten 50, 5005 Bergen, Norway; 6: corresponding author, [tanja.kogel@hi.no](mailto:tanja.kogel@hi.no).

## Chemical solutions and filters

Table S1 Chemical solutions used in the experiments

| Solution | Procedure |
| --- | --- |
| Fenton’s reagent | 30 % H_2_O_2_ and an iron catalyst solution prepared with 10 g iron (II) sulfate heptahydrate in 500 ml Milli-Q water. The iron catalyst solution and H_2_O_2_ were added into glass beakers in turn to the volume ratio of 1:2.5 (Yan, Zhao et al. 2020). |
|  |  |
| H_2_O_2_ 15 % and HNO_3_ 5% mixture | For 400 ml solution, 170 ml milli-Q water was added to a glass bottle followed by 200 ml of 30 % H_2_O_2_. Next 30 ml of HNO_3_ 65 % was added. The solution was filtered through Whatman™ GF/C. |
|  |  |
| 20 % KOH solution | KOH pellet was dissolved in Milli-Q water and filtrated through Whatman™ GF/C |
|  |  |
| 10 % KOH solution | Diluting 20 % KOH with Milli-Q water and filtrated through Whatman™ GF/C. |
|  |  |
| Tris HCl 1 M, pH 8 buffer | For 1 L, 121.14 g of Tris (tris(hydroxymethyl)aminomethane) was dissolved in 800 ml of Milli-Q water. PH 8 was titrated using concentrated HCl. Milli-Q water to 1 L was added, filtered through Whatman™ GF/C. |
|  |  |
| Creon solution | 0.08 g Creon particles / g sample were dissolved in 1 M Tris (HCl) pH 8 (von Friesen, Granberg et al. 2019). |
|  |  |
| NaOAc (C_2_H_3_NaO_2_) 1 M, pH 5 buffer | For 1 L, 82.03 g NaOAc (anhydrous) was dissolved in 800 ml Milli-Q water. PH 5 was titrated using concentrated acetic acid (H_3_CCOOH). Milli-Q water to 1 L was added and filtered through Whatman™ GF/C. |
|  |  |
| Viscozyme® L solution | 0.5 ml Viscozyme® L per sample were added to 12 ml of NaOAc buffer and filtered through Whatman™ GF/C. |
|  |  |
| Cellulase TXL solution | 10 ml Cellulase TXL per sample were added to 50 ml NaOAc buffer and filtered through Whatman™ GF/C. |

Table S2 Filters used in the experiments

| Filter | Pore size (µm) |
| --- | --- |
| Whatman™ GF/D | 2.7 |
| Whatman™ Cellulose Nitrate Membrane | 5 |
| Whatman™ GF/C | 1.2 |
| Whatman™ GF/F | 0.7 |
| PTFE filter | 5 |
| ROBU® 50 ml crucibles Fine (ASTM E-128-99) | 4.5-5 |

## Diet

Composition: Wheat, barley, wheatfeed, dehulled extracted toasted soya, soya protein concentrate, minerals, soya bean oil, vitamins, whey powder, amino acids.

Table S3 Analytical constituents of RMI (E) SQC feed

| Nutrient | Total |
| --- | --- |
| Crude fat | 2.5 % |
| Crude protein | 14.4 % |
| Crude fiber | 4.4 % |
| Crude ash | 5.4 % |
| Lysine | 0.57 % |
| Methionine | 0.26 % |
| Calcium | 0.74 % |
| Phosphorous | 0.51 % |
| Sodium | 0.24 % |
| Magnesium | 0.21 % |
| Copper | 12 mg/kg |

## Optimized protocol

0.1 g and 0.3 g of feces and feed samples respectively, were incubated for 24 h at 40 °C with 30 ml of the mixture of H_2_O_2_ 15 % and HNO_3_ 5 %. The digestates were then vacuum filtered through 5 µm cellulase nitrate filters. 30 ml of 10 % KOH solution was added to the flasks containing the filter and incubated again under the same conditions. The cellulase nitrate filter can be fully dissolved in KOH. The samples were then filtered through PTFE filter (5 μm). The PTFE filters were then prepared for the digestion with Viscozyme® L and incubated for 24 h at 40 °C. Lastly, the digestates were filtered through Whatman™ GF/C (1.2 μm) and placed in an incubator at 56 °C to dry prior py-GC/MS-Orbitrap™ analysis.

Protocol 3 included important washing steps, in which the Erlenmeyer flasks and the filters in each digestion step were washed with 0.01 % (m/m) Tween®-20, ethanol-water (1:1, v/v), and water. In the final step prior to transferring the samples into Frontier pyrolizer cups, Whatman™ GF/C filters were washed with pre-filtered 0.01 % (m/m) Tween®-20, ethanol-water (1:1, v/v), and 96 % ethanol. Lastly, few drops of 20 % KOH were added directly on the filter to ease the filtration.

## FTIR analysis of the remained fecal material after acidic and alkaline treatment.

After the acidic oxidative digestion followed by an alkaline digestion step a white fibrous substance remained on the filter. The residual material on the filter was identified as cellulose by ATR-IR analyses (Figure S1).


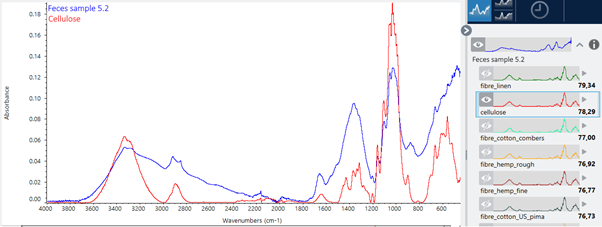


Figure S1. ATR-IR spectrum of a fecal sample after acidic and alkaline digestion

Following further treatment with Creon® the substance appeared mostly homogeneous (Figure *S2*). By visual observation there was a lot more of the material left from fecal samples than from the diet, suggesting that this substance was to a lesser extent being metabolized by the rat but rather concentrated in the fecal material.


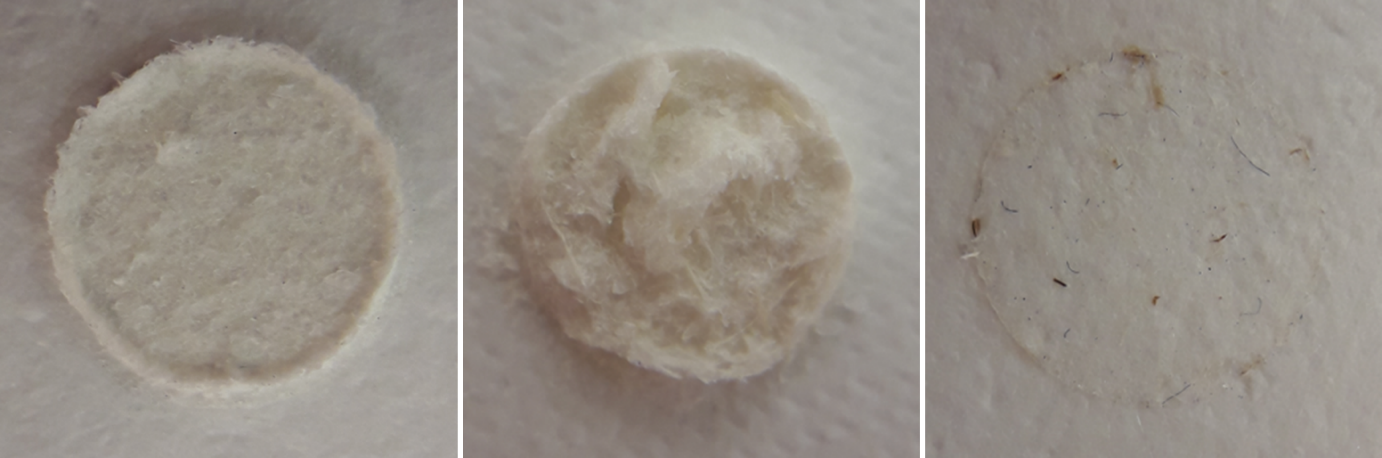


**A**

**B**

**C**

Figure S2. White homogenous substance present on the filters after treating samples with i) H_2_O_2_ (15 %) and HNO_3_ (5 %), ii) KOH (10 %) and iii) Creon® or Viscozyme® L. The amount of substance from the diet (A) was visually significantly less than from fecal (B) after using Creon®. The samples after treatment with Viscozyme® L is shown in the third image (C).

A Nicolet™ Summit PRO FTIR spectrometer equipped with an Everest™ ATR accessory monolithic ATR crystal was used to collect the spectra from 4000 cm^−1^ to 440 cm^−1^. For each sample 16 co-added scans with spectral resolution of 4 cm^-1^ were collected and 32 pre-recorded background scans were used for correction. Spectra were processed in the OMNIC Paradigm Desktop software (Thermo Scientific™) and compared to spectra of known compounds and polymers in commercial libraries (Thermo Scientific™), open-source libraries (simple-plastics.eu; (Primpke, Wirth et al. 2018) and in-house libraries of plastic and natural polymers.

## Recovery rate

Table S4 Recovery results of PA particles added to each triplicate of feed and feces

| Sample type | MPs added (mg/g) | MPs recovered (mg/g) | Recovery (%) | Average of recovery (%) | SEM |
| --- | --- | --- | --- | --- | --- |
| Feed 1 | 0.97 | 1.62 | 167 | 105 | 31 |
| Feed 2 | 0.98 | 0.73 | 74 |  |  |
| Feed 3 | 1 | 0.74 | 74 |  |  |
| Feces 1 | 15.76 | 15.2 | 96 | 88 | 14.42 |
| Feces 2 | 13.99 | 15.3 | 108 |  |  |
| Feces 3 | 15.42 | 9.35 | 60 |  |  |

Table S5 Recovery results of PE particles added to each triplicate of feed and feces

| Sample type | MPs added (mg/g) | MPs recovered (mg/g) | Recovery (%) | Average of recovery (%) | SEM |
| --- | --- | --- | --- | --- | --- |
| Feed 1 | 0.97 | 2.48 | 255 | 121.67 | 66.97 |
| Feed 2 | 0.98 | 0.65 | 66 |  |  |
| Feed 3 | 1.33 | 0.59 | 44 |  |  |
| Feces 1 | 19.7 | 25 | 126 | 82.67 | 28.10 |
| Feces 2 | 14.76 | 13.6 | 92 |  |  |
| Feces 3 | 14.61 | 4.5 | 30 |  |  |


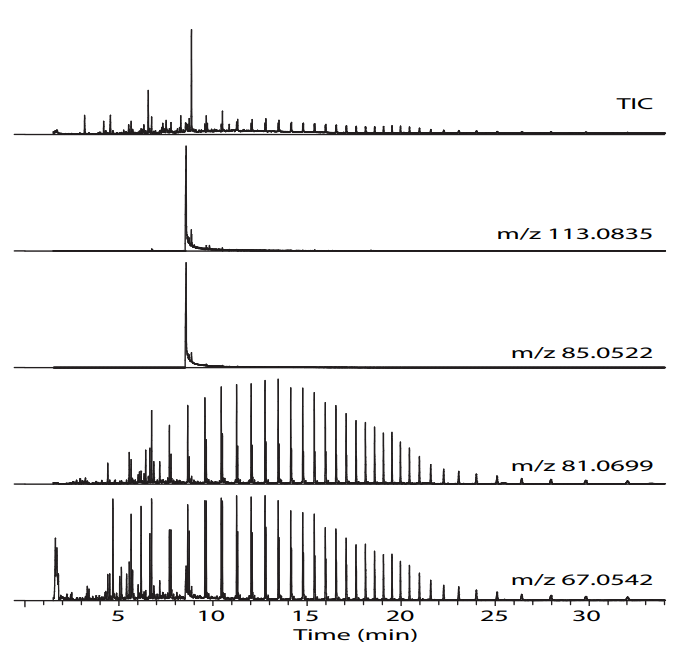


Figure S3. Pyrograms of digested feces from rats exposed to PA and PE. Total ion chromatogram (window 1) and selective ions for quantification and confirmation of PA (window 2-3) and PE (window 4-5).


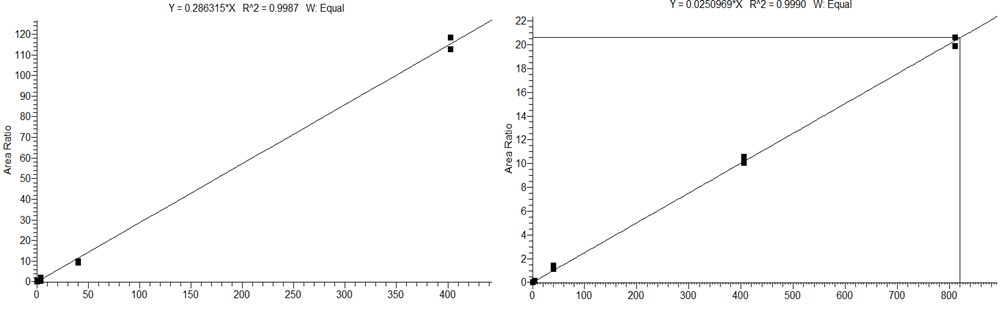


Figure S4. Calibration curves for PA-6 (left) and PE (right) used for the control samples. Area ratio was calculated against deuterated internal standard (PS-D8).

## References

Primpke, S., M. Wirth, C. Lorenz and G. Gerdts (2018). "Reference database design for the automated analysis of microplastic samples based on Fourier transform infrared (FTIR) spectroscopy." Analytical and bioanalytical chemistry **410**(21): 5131-5141.

von Friesen, L. W., M. E. Granberg, M. Hassellöv, G. W. Gabrielsen and K. Magnusson (2019). "An efficient and gentle enzymatic digestion protocol for the extraction of microplastics from bivalve tissue." Marine pollution bulletin **142**: 129-134.

Yan, Z., H. Zhao, Y. Zhao, Q. Zhu, R. Qiao, H. Ren and Y. Zhang (2020). "An efficient method for extracting microplastics from feces of different species." Journal of hazardous materials **384**: 121489.
